# Association between serum copper levels and cervical cancer risk: a meta-analysis

**DOI:** 10.1042/BSR20180161

**Published:** 2018-07-06

**Authors:** Min Zhang, Min Shi, Yan Zhao

**Affiliations:** Department of Obstetrics and Gynecology, Shandong Provincial Hospital Affiliated to Shandong University, Jinan 250021, China

**Keywords:** Copper level, Cervical cancer, Meta-analysis, Serum

## Abstract

Whether serum copper levels were higher in patients with cervical cancer than that in controls was controversial. Hence, we conducted the present study to explore the relationship between serum copper levels and cervical cancer. We searched PubMed, WanFang, and China National Knowledge Internet (CNKI) for relevant studies before November 30, 2017. Standardized mean difference (SMD) and 95% confidence interval (CI) were used to combine results across studies using the random-effect model. A total of 14 publications involving 747 patients with cervical cancer and 1014 controls were eligible through inclusion criteria. In comparison with controls, serum copper levels were significantly higher in patients with cervical cancer [summary SMD = 1.35; 95%CI: 0.10–2.59], with significant heterogeneity (*I^2^* = 98.8%; *P*<0.001) was found. Significant association was also found among Asian populations [summary SMD = 1.39; 95%CI: 0.06–2.71]. The association was positive in subgroup analysis of population-based case–control studies (PBCC) [summary SMD = 1.64; 95%CI: 0.02–3.34], but not in hospital-based case–control studies (HBCC). Through a sensitivity analysis, we did not identify any single study to strongly influence the results of our serum copper levels and cervical cancer risk. No publication bias was found in our analysis. In conclusion, our study provided significant evidence of higher serum copper levels in patients with cervical cancer than in controls, suggesting that serum copper exposure was a risk factor on cervical cancer.

## Introduction

Cervical cancer is a malignant tumor derived from cervical cells. Cervical cancer is the second most common cancer among women worldwide [1], accounting for approximately 527,600 new female cancer cases and 265,700 deaths worldwide in 2012 [2]. In the current society, especially in the developing countries, the prognosis of cervical cancer patients remains poor [3,4]. A variety of factors had been reported to be associated with the development of cervical cancer, such as human papillomavirus (HPV) infection and genetics factors [5,6]. Furthermore, micronutrients like serum selenium levels also played an important role in the cervical cancer risk [7].

Copper is one of the trace elements in our bodies, and to date, a subset of investigators have researched the associations between serum copper levels and cervical cancer risks, and a number of epidemiologic studies have been published in this field. Eleven studies [8–18] suggested that serum copper levels are higher in patients with cervical cancer compared with that in controls, while one article [19] had reported the lack of significant association. However, two publications [20,21] indicated that it was significantly lower in patients with cervical cancer relative to controls about serum copper levels. Therefore, the current meta-analysis was to explore whether serum copper levels in patients with cervical cancer are higher than in controls. We also evaluated the potential heterogeneities between individual studies.

## Methods

### Study selection

Studies were identified through searching the databases of PubMed, WanFang, and China National Knowledge Internet (CNKI) up to November 30, 2017. The following search terms were used: ‘serum’ AND ‘copper’ OR ‘Cu’ AND ‘cervical cancer’ OR ‘cervical carcinoma’ without restrictions. Reference lists and the studies retrieved were also examined to find any additional study potentially unidentified. Two investigators independently performed the article search and reviewed the relevant references.

Inclusion criteria were the following: having a prospective cohort design or a case–control design or a cross-sectional study; evaluating the association between serum copper levels and cervical cancer risk; reported mean and standard deviation (SD) of copper levels both in cervical cancer patients and controls; studies published in English language or Chinese language. The selection process was independently performed by two authors and retrieved articles were examined.

### Data extraction and study quality

Data were abstracted from each identified study by using a standardized extraction form. The following information was collected: first author name; publication years; study design; country; number of cervical cancer cases and controls; age range or mean age of the cases; mean and SD of copper levels both in cervical cancer patients and controls. This process was independently performed by two authors (M.Z. and M.S.) and discrepancies were discussed and resolved by consensus.

The quality of studies was assessed by the Newcastle–Ottawa Scale (NOS) [22]. The NOS ranges from 0 to 9 stars. The studies with ≥ 6 stars were considered as high quality [23].

### Statistical analysis

The relationship between serum copper levels and cervical cancer risk was pooled using standardized mean difference (SMD) with 95% confidence interval (CI). A random-effects model for the current meta-analysis was used [24]. Heterogeneity among included studies was assessed by the *I^2^* of Higgins et al. [25], where *I^*2*^* describes the proportion of total variation attributable to between-study heterogeneity as opposed to random error or chance, and *I^*2*^-*values ≤25, ≤50, ≤75, and >75% indicated no, little, moderate, and significant heterogeneity respectively [26]. We used meta-regression to assess the potential of important covariates to exert substantial impacts on between-study heterogeneity. Egger’s regression asymmetry test [27] and Begg’s funnel plot [28] were used to visually examine publication bias on study outcome. A sensitivity analysis by exclusion of one study at a time was performed to assess the stability of results and potential sources of heterogeneity [29]. We used STATA version 12.0 (Stata Corporation, College Station, TX, U.S.A.) for the meta-analysis. *P*≤0.05 (two-tailed) indicates statistical significance.

## Results

### Search results and study characteristics

**Figure 1:**
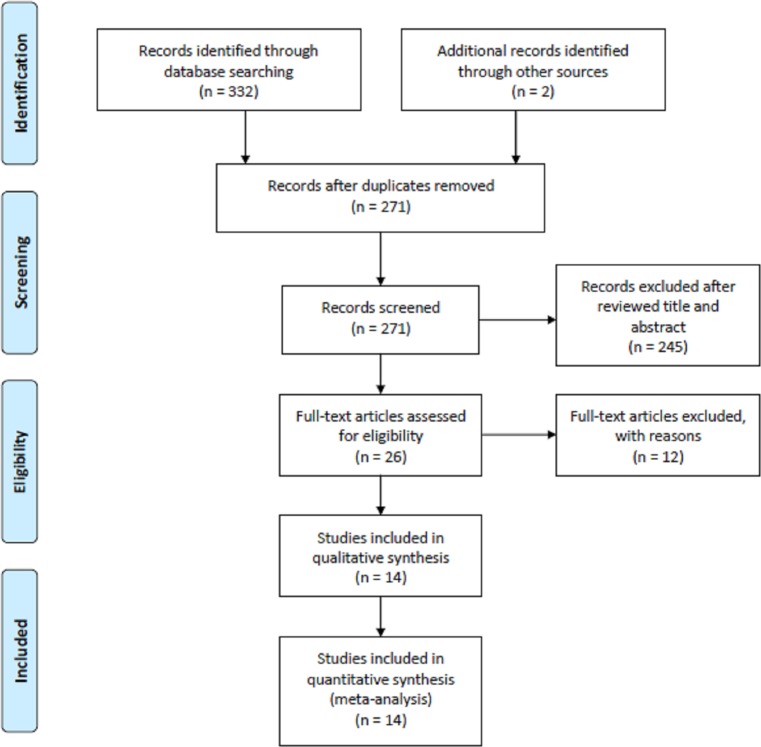
Flow diagram of the literature search

The process of identification and study selection is summarized in Figure 1. Among the initial 332 articles screened through databases of PubMed, WanFang, and China National Knowledge Internet (CNKI) searching and two additional records identified through other sources, there are 271 articles reviewed the title and abstract while excluding the duplications from different databases. A total of 245 articles out of 271 articles were accepted when screened on the basis of title and abstract, 26 articles were screened by reading full-texts. Twelve studies were excluded after a full-text examination for the following reasons: six articles were reviews, two articles did not report mean or SD of serum copper levels, three articles were animal studies, one article was letter to the editor. Hence, 14 articles [8–21] were eligible to be included in the analysis comprising 747 patients with cervical cancer and 1014 controls. All the included studies were case–control design. Eight studies were carried out from China, three from India, one from Turkey, one from U.K., and one from Korea. In quality assessment, the average quality scores were 7.07, and all included studies had a score of greater or equal to 6. The characteristics of included studies are listed in Table 1.

**Table 1:** Characteristics of the included studies about serum copper levels and cervical cancer risk

Study, year	Country	Age (range or Mean ± SD)	Study type	Study quality scores	Cervical cancer cases		Controls	
					*n*	Serum copper: mean ± SD	*n*	Serum copper: mean ± SD
Arumanayagam et al., 1993 [8]	China	58.7± 1.83	HBCC	7	57	19.1 ± 0.55 (μmol/l)	24	18.2 ± 0.75 (μmol/l)
Cetinkaya et al., 1988 [9]	Turkey	NA	PBCC	7	9	1.69 ± 0.6 (μg/ml	20	0.44 ± 0.46 (μg/ml)
Chen et al., 1990 [10]	China	25–70	HBCC	7	99	117.1 ± 14.6 (μg/ml)	50	109.4 ± 17.4 (μg/ml)
Cunzhi et al., 2003 [11]	China	30–65	PBCC	6	40	19 ± 7 (μmol/l)	50	14 ± 4 (μmol/l)
Fu et al., 2009 [12]	China	31–70	PBCC	7	74	18.96 ± 3.25 (μmol/l)	180	15.78 ± 3.88 (μmol/l)
Grail et al., 1986 [13]	U.K.	25–60	PBCC	8	24	1.47 ± 0.26 (mg/l)	21	1.25 ± 0.16 (mg/l)
Kim et al., 2003 [19]	Korea	35–74	HBCC	7	36	101.6 ± 5.25 (μg/dl)	44	101.5 ± 5.47 (μg/dl)
Naidu et al., 2007 [14]	India	25–65	PBCC	8	30	117.4 ± 12.26 (μg%)	30	109.7 ± 10.85 (μg%)
Ramteke et al., 2015 [15]	India	35–75	PBCC	8	50	156.9 ± 3.4 (μg/dl)	50	107.2 ± 1.79 (μg/dl)
Subramanyam et al., 2013 [20]	India	30–75	PBCC	7	104	78.15 ± 2.8 (μg/dl)	50	98.76 ± 2.43 (μg/dl)
Wang et al., 2010 [16]	China	25–60	PBCC	7	41	14.23 ± 3.15 (μmol/l)	260	12.73 ± 2.56 (μmol/l)
Yu et al., 2016 [21]	China	36–65	PBCC	7	70	12.39 ± 2.1 (μmol/l)	150	48.74 ± 6.5 (μmol/l)
Zhang et al., 1996 [17]	China	32–61	PBCC	6	55	24.93 ± 6.05 (μmol/l)	35	19.31 ± 2.46 (μmol/l)
Zhang et al., 2015 [18]	China	32–70	PBCC	7	58	50.9 ± 4.5 (μmol/l)	50	10.3 ± 4 (μmol/l)

Abbreviations: HBCC, hospital-based case–control study; PBCC, population-based case–control study.

### Serum copper levels and cervical cancer risk

**Figure 2:**
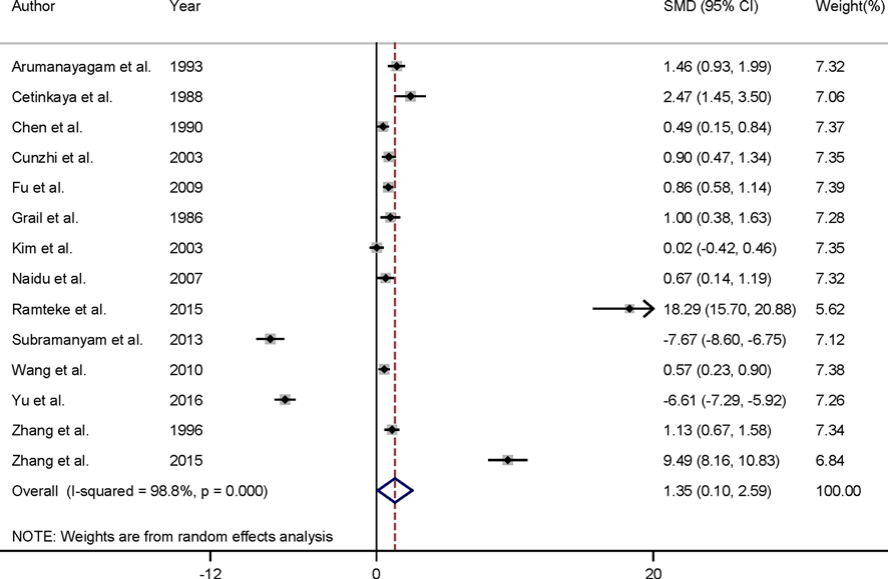
Forest plot of SMD with corresponding 95%CI of studies about serum copper levels and cervical cancer risk

Forest plots of the association between serum copper levels and cervical cancer risk across all studies are shown in Figure 2. About the 14 included studies, pooled results suggested that patients with cervical cancer had significantly higher serum copper levels than that in controls [summary SMD = 1.35; 95%CI: 0.10–2.59; *I*^2^ = 98.8%; *P*_for heterogeneity_< 0.001].

Within subgroup analysis by geographic location, we only pooled the result for Asian populations because 13 of the 14 articles were from Asia. However, only one article comes from Europe, and the result was not pooled. The relationship was significant among Asian populations [summary SMD = 1.39; 95%CI: 0.06–2.71; *I*^2^ = 98.9%; *P*_for heterogeneity_<0.001]. When we conducted the subgroup analysis by HBCC or PBCC, the association was only significant in the group of PBCC [summary SMD = 1.64; 95%CI: 0.02–3.34; *I*^2^ = 99.1%; *P_for heterogeneity_*< 0.001], but not in HBCC [summary SMD = 0.64; 95%CI: −0.09–1.38; *I*^2^ = 88.3%; *P _for heterogeneity_*< 0.001].

### Sources of heterogeneity and meta-regression

As seen in the pooled result, we found evidence of significant heterogeneity in our pooled result summary (*I^2^*= 98.8%; *P*<0.001). Thus, univariate meta-regression was performed to explore whether the reason of heterogeneity was associated with covariates of publication year, case numbers, and geographic location. No significant contribution to between-study heterogeneity was found in this analysis (*P*=0.172; 0.231, 0.198 for publication year, case numbers, and geographic location respectively).

### Sensitivity analysis and publication bias

**Figure 3:**
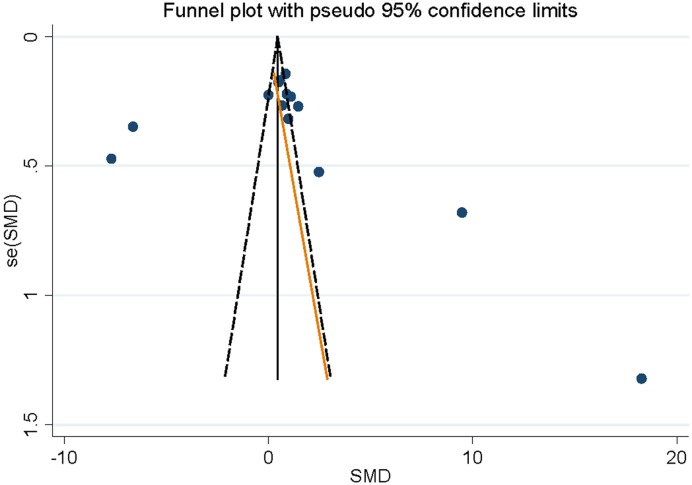
Begg’s funnel plot about the association between serum copper levels and cervical cancer risk

Sensitivity analysis conducted by excluding one study at the time revealed that no single study had essential effect on the whole result. No evidence of significant publication bias was confirmed by Egger’s test (*P*=0.722) and funnel plot (Figure 3).

## Discussion

In the present study, we explored the relationship between serum copper levels and cervical cancer risk. The results found that serum copper levels in patients with cervical cancer were significantly higher than that in controls. Through our subgroup analysis, we further found significant relationship among Asian population and the subgroup analysis of PBCC.

Copper is an important mineral that plays an important role in various biochemical reactions as a cofactor for superoxide dismutase (SOD). This enzyme plays an important role in protecting the body against free radicals [30]. The carcinogenic activity of copper is believed to be related to the formation of reactive oxygen species that damage the DNA line and the initiation of tumor angiogenesis [31]. Similar to this, our findings of raised serum copper levels are very well correlated with the above mentioned mechanism.

As seen in Figure 2, in our whole-pooled result, significant between-study heterogeneity was appeared (*I^2^*= 98.8%; *P _for_*_heterogeneity_< 0.001), which is common in meta-analysis [32]. We then performed meta-regression to assess this high heterogeneity with covariates of publication year, case numbers, and geographic location. As a result, all the above mentioned factors were not found to significantly contribute to heterogeneity. To further explore the between-study heterogeneity among location and sources of controls, subgroup analyses were performed. However, between-study heterogeneity was evident in certain subgroups.

Our meta-analysis has the following advantages. First, we performed the first meta-analysis to expound the risk factor of serum copper levels in cervical cancer patients. Second, according to our final pooled analysis for each individual study, larger numbers of cervical cancer patients and controls were included. This may strengthen the accurate comparisons between serum copper levels and cervical cancer risk. Third, subgroup analyses were performed to find the detailed results for location and sources of controls, and we observed that the association was significant in the subgroup analysis of Asian populations. The same conclusion was acquired in the subgroup analysis of PBCC. Fourth, no publication bias was found due to Egger’s test and funnel plot, which indicated that our results were stable across included studies.

Although, we attempted to evaluate the association between serum copper levels and cervical cancer risk more comprehensively, some limitations still existed. First, all the included researches were case–control studies. Although some recollection bias and selection bias would be appeared in case–control studies, they were an important method in observational studies. Nevertheless, more studies with prospective design are needed in the future. Second, heterogeneity among studies was relatively large in our meta-analysis on account of the absence of valuable information, which might reduce the persuasive power of the pooled estimation. However, we used a random-effect model to combine the results. As we all know, random-effect model had wider range about 95%CI than fixed-effect model, and could obtain more accurate results. Furthermore, sensitivity analysis by exclusion of one study at the time was performed to assess the stability of results and potential sources of heterogeneity. No study had essential effect to the significant between-study heterogeneity and the whole result. Finally, 13 of the 14 included studies were from Asia; however, only one included study was from Europe, and thus, additional studies from other countries are required to further define associations between geographic location and cervical cancer risk.

In summary, our study provided significant evidence of higher serum copper levels in patients with cervical cancer than in controls, suggesting that serum copper exposure was a risk factor on cervical cancer.

## Abbreviations

CI: confidence interval
CNKI: China National Knowledge Internet
HBCC: hospital-based case–control study
NOS: Newcastle–Ottawa Scale
PBCC: population-based case–control study
SD: standard deviation
SMD: standardized mean difference
